# Real-Time Tracking of Object Melting Based on Enhanced DeepLab *v*3+ Network

**DOI:** 10.1155/2022/2309317

**Published:** 2022-03-30

**Authors:** Tian-yu Jiang, Feng-lan Ju, Ya-xun Dai, Jie Li, Yi-fan Li, Yun-jie Bai, Ze-qian Cui, Zheng-han Xu, Zun-Qian Zhang

**Affiliations:** ^1^Hebei Engineering Research Center for the Intelligentization of Iron Ore Optimization and Ironmaking Raw Materials Preparation Processes, North China University of Science and Technology, Tangshan, Hebei 063210, China; ^2^Hebei Key Laboratory of Data Science and Application, North China University of Science and Technology, Tangshan, Hebei 063210, China; ^3^Key Laboratory of Engineering Computing, North China University of Science and Technology, Tangshan, Hebei 063210, China; ^4^Tangshan Intelligent Industry and Image Processing Technology Innovation Center, North China University of Science and Technology, Tangshan, Hebei 063210, China; ^5^College of Metallurgy and Energy, North China University of Science and Technology, Tangshan, Hebei 063210, China

## Abstract

In order to reveal the dissolution behavior of iron tailings in blast furnace slag, the main component of iron tailings, SiO_2_, was used for research. Aiming at the problem of information loss and inaccurate extraction of tracking molten SiO_2_ particles in high temperature, a method based on the improved DeepLab *v*3+ network was proposed to track, segment, and extract small object particles in real time. First, by improving the decoding layer of the DeepLab *v*3+ network, construct dense ASPP (atrous spatial pyramid pooling) modules with different dilation rates to optimize feature extraction, increase the shallow convolution of the backbone network, and merge it into the upper convolution decoding part to increase detailed capture. Secondly, integrate the lightweight network MobileNet v3 to reduce network parameters, further speed up image detection, and reduce the memory usage to achieve real-time image segmentation and adapt to low-level configuration hardware. Finally, improve the expression of the loss function for the binary classification model of small object in this paper, combining the advantages of the Dice Loss binary classification segmentation and the Focal Loss balance of positive and negative samples, solving the problem of unbalanced dataset caused by the small proportion of positive samples. Experimental results show that MIoU (mean intersection over union) of the proposed model for small object segmentation is 6% higher than that of the original model, the overall MIoU is increased by 3%, and the execution time and memory consumption are only half of the original model, which can be well applied to real-time tracking and segmentation of small particles.

## 1. Introduction

With the rapid development of computer vision, image segmentation technology, as a key field of graphics and image processing, has gradually stepped into the development of new concepts [1]. Image segmentation refers to the process of planning pixel values with the same attributes into the same label by using the nonlinear relationship between the difference and correlation of different pixel values. Image segmentation can provide concise and reliable image feature information and then effectively improve the processing efficiency of subsequent visual tasks, which is of great significance. In the fields of unmanned driving, medical impact observation, satellite remote sensing, etc., different methods are used to adapt the internal chip and logic algorithm according to the actual requirements, so as to meet the requirements of different segmentation tasks.

With the gradual development of convolutional neural network [2] and the proposal of image semantic segmentation [3], image segmentation technology has been greatly improved. Image semantic segmentation can accurately locate the image content and fully present the semantic features of the region composed of the same attribute pixels by predicting and classifying the pixels. Due to the high complexity of image semantic segmentation, enhancing the representation ability of image pixels and improving the information utilization rate of multilayer convolution are the key directions of segmentation. In 2015, J. Long [4] and K. Simonyan [5] proposed Full Convolutional Network for image segmentation, whose core idea is to remove the fully connected layers of the network structure and complete prediction through the feature map of the final convolutional layer. This method has promoted the development of semantic segmentation.

Image spatial information is to strengthen the relationship between different image channels, and the correlation can be adjusted through this spatial information. Therefore, the method of dilated convolution was introduced. Through this method, DeepLab v1 [6] solved a series of information loss problems caused by convolution operations. However, this method has the following problems: (1) reduced feature resolution, (2) the existence of multiscale objects, and (3) decreased spatial accuracy due to spatial invariability of dilated convolution and so on. Further, two methods, DeepLab v2 [7] and DeepLab v3 [8], were introduced. (1) The same model uses shared weight, which is suitable for multiscale input. (2) The feature response of large-scale input preserves the details of small objects. (3) The method transforms the input into multiscale through the Laplacian pyramid. The DeepLab *v*3+ network structure used in this paper combines the above advantages, has a simple and effective encoding-decoding structure and ASPP module that aggregates multiscale features, and has achieved excellent results in multiple public datasets [9].

Since this article is research on industrial manufacturing technology, the cameras in different factories are different, and the pixels for capturing images are also different. This network framework can reduce the impact of resolution very well. The framework does a good job of reducing the memory usage of the GPU, only using multiscale inputs in the final prediction. Therefore, this paper selects the DeepLab *v*3+ network for in-depth research. At present, the model also requires high-performance computers, which is not suitable for factory equipment, and the tracking and recognition accuracy of small objects are not high, so this cannot be put into production. Therefore, it is necessary to improve the model.

In order to improve the tracking accuracy of small objects, solve the problem of uneven positive and negative samples, and reduce the requirements of computer performance to meet the requirements of factories, this paper plans to conduct an in-depth study on the DeepLab *v*3+ model to improve the remaining shortcomings of the model structure. In DeepLab *v*3+ model coding stage, context information is aggregated through ASPP, but the small object segmentation has the disadvantage of low accuracy and lack of spatial correlation. In the decoding stage, only one of the multistage shallow features on the backbone network is fused, resulting in partial loss of effective information, in segmentation discontinuity, and in rough segmentation boundary. Therefore, in this paper, the network architecture is modified to increase the feature layer fusion in the decoding stage and then strengthen the feature pixel learning. Combining with the lightweight network, the problem of redundant network parameters and high hardware requirements is improved, and the form of loss function is modified to adopt to the problem of binary classification and uneven distribution of positive and negative samples in this paper.

The first part of this article introduces the DeepLab *v*3+ network framework used in this article by introducing the significance of computer to image segmentation. The second part briefly introduces the related work and the latest research of the network framework used in this article. The third part deeply analyses the advantages and disadvantages of the DeepLab *v*3+ framework as well as the development history and performance of the MobileNet v3 network, paving the way for improvement. The fourth part describes the details of the author's improvement of network architecture. The fifth part combines experimental data to conduct a conclusion analysis and prove the advantages of the algorithm in this article. The sixth part is a summary description of this paper.

## 2. Related Work

Image segmentation algorithms have developed rapidly in recent years, and many researchers have improved and optimized the deep learning framework of semantic segmentation algorithms and then applied them to daily life and industrial manufacturing. For DeepLab *v*3+ improvement research direction, Baheti B [10] focused his research on India Driving Dataset which contains data from unstructured traffic scenario and modified the DeepLab *v*3+ framework by using lower atrous rates in ASPP module for dense traffic prediction. D. Wu [11]et al. used the framework of ResNet-101 to develop the DeepLab *v*3+ semantic segmentation model to segment the data frames collected from 70 video clips of different cows. An ensemble method for crack detection is based on convolutional neural networks, of which DeepLab *v*3+ was found to be reliable and widely applicable for crack detection. Among the quantitative indicators, the prediction value of crack length has the lowest relative error rate. A. Ji [12] proposed an integrated approach based on the convolutional neural network for crack detection, in which DeepLab *v*3+ was found to be reliable and widely applicable or crack detection. Among various quantitative indicators, the relative error rate of the predicted value of crack length is the lowest. S. Cheng [13] used the DeepLab *v*3+ to segment smoke images. U. Verma [14]et al. used DeepLab *v*3+ for river identification and width measurement. For other algorithms to split the direction, K. Iyer [15] designed a convolutional neural network AngioNet for vessel segmentation in X-ray angiography images. The best performance was obtained using Deeplabv3+. Wang [16]proposes a dense FCN (fully convolutional network) which combines dense network with FCN model and achieves good semantic segmentation effect. Q. Liu [17]proposes a multilevel similarity model under a Siamese framework for robust thermal infrared object tracking. He designed a simple while effective relative entropy based ensemble subnetwork to integrate the semantic and structural similarities. The proposed algorithm performs favorably against the state-of-the-art methods. Yuan [18]proposes an effective self-supervised learning-based tracker in a deep correlation framework which achieves competitive tracking performance contrasted to state-of-the-art supervised and unsupervised tracking methods on standard evaluation benchmarks. For picture prediction direction, Huang [19]proposes a novel network structure, namely, Kernel-Sharing Atrous Convolution, where branches with different receptive fields share the same kernel; i.e., let a single kernel ‘see' the input feature maps more than once with different receptive fields. Li [20] proposes a deep learning scheme to achieve fine extraction of image water bodies. The process includes multiscale feature perception splitting of images, a restructured deep learning network model, multiscale joint prediction, and postprocessing optimization performed by a fully connected conditional random field. For small objects, Yang [21] proposes a real-time segmentation model that creates a narrow deep network and constructs a synthetic dataset by inserting additional small objects in training images. An average 2% MIoU improvement is obtained on small objects. For the modification of the loss function, big data can solve most data problems [22], assembling algorithms to adapt to specific problems [23].

Therefore, this paper uses this framework to study the online tracking of small target melting and obtains a network model with high accuracy and low training fluctuation.

## 3. Related Theory

### 3.1. Traditional DeepLab *v*3+ Network

**Figure 1:**
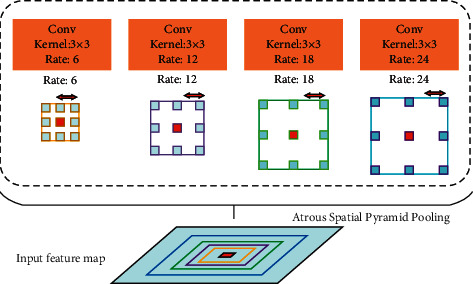
Atrous spatial pyramid pooling.

Taking the residual model as the underlying network and adding an encoder-decoder structure, DeepLab *v*3+ model is an improvement of DeepLab v3 model and belongs to a typical Dilated Fully Connected Network framework. The network framework ResNet [24] or Xception [25] was used for feature extraction of the input images, and then ASPP was used, as shown in Figure 1, mainly to introduce multiscale information and to fuse image features through image dilated convolution to reduce the loss of image feature. ASPP is designed to capture multiscale information, which is critical to segmentation accuracy. Among them, rate (*r*) controls the size of the receptive field, and the greater the *r*, the greater the receptive field [26].

**Figure 2:**
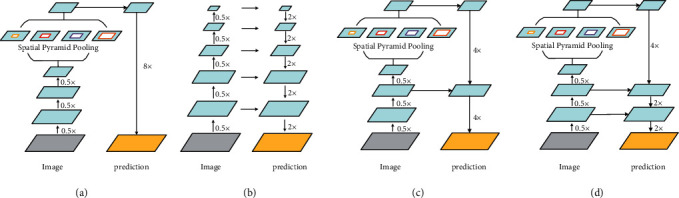
(a) DeepLabv3 model diagram; (b) encoding and decoding methods; (c) improved DeepLab *v*3+ model diagram influenced by decoding ideas; (d) the model structure realized in this paper.

As shown in Figure 2(b), the DeepLabv3+ model borrowed the encoder-decoder structure and introduced a new decoder module. First, use bilinear interpolation to quadruple the feature obtained by the encoder, and then connect with the low-level feature of the corresponding size in the encoder. In order to prevent the high-level feature obtained by the Encoder from being weakened, 1 × 1 volume convolution was used to reduce the dimensionality of the low-level feature. After the two features being connected, 3 × 3 volume convolution was used to further fuse. Finally, bilinear interpolation was performed to obtain a segmentation prediction of the same size as the original image.

The modified Xception is attempted in DeepLab *v*3+ model. The Xception network mainly uses depthwise separable convolution [27], which makes the calculation of Xception lower. (1) Add more layers; (2) replace all the max pool layer with depthwise separable convolutions with step size of 2, which can be changed into dilated convolution. (3) Add batch standardization and ReLu activation functions after 3×3 volume depthwise convolution.

### 3.2. MobileNet v3

With the MobileNet structure proposed [28], the lightweight network framework has developed rapidly. As the backbone, MobileNet is three times faster than the Vgg [29] network. MobileNet v2 [30] added the idea of residual model and the inverted residual structure to prevent vanishing gradient. The concept of bottleneck was designed to reduce input and output parameters and compress the model structure again. The ReLu behind the pointwise convolution was replaced with a linear function, and the output result was 0 after reducing the number of nodes. This paper introduces the MobileNetv3 model. First, the network architecture is based on MnasNet [31] implemented by NAS, which is better than MobileNet v2. The MobileNet v3 model combines the depthwise separable convolution of MobileNet v1 with the inverted residual structure of MobileNet v2 with linear bottlenecks. Secondly, a lightweight attention model based on squeeze and excitation structure is introduced to weight different channels, increasing the important channel weights and decreasing the unimportant channel weights. Third, the activation function is improved, using a new activation function h-swish instead of ReLu to significantly improve the accuracy of neural network. In network structure search, two technologies are combined: (1) platform-aware NAS [32] is used to optimize each block by using search the network when the calculations and parameters are limited. (2) NetAdapt [33] is used to fine-tune the number of convolution kernels in each layer of the network layer after each module is determined.

## 4. Deep Learning Network Construction and Improvement

### 4.1. DeepLab v3+ Network Improvement

In this paper, the author uses DeepLab *v*3+ algorithm as a semantic segmentation method based on fully supervised learning, using deep convolutional neural networks to achieve target segmentation and using the dilated convolution to balance the accuracy and time consumption through.

Since semantic segmentation is an end-to-end network structure, upsampling of the prediction images obtained by convolutional neural networks is required. DeepLab *v*3+ model is improved for upsampling. As shown in Figure 2(c), it divides 8-fold upsampling into two 4-fold upsampling operations, i.e., 16-fold upsampling, and then goes through a refinement operation of 3*∗*3 convolution to obtain high accuracy and fast speed, which combines the advantages of residual model and gathers high-level and low-level information. Since the volume of SiO_2_ gradually becomes smaller during the melting process, the segmentation accuracy of this network for small targets is not high and the phenomenon of loss exists in this network. Therefore, inspired by the YOLO [34] target detection algorithm, as shown in Figure 2(d), this paper divides the above 16-fold upsampling into two 2-fold operations and one 4-fold operation, combines image of the first convolution of the original image, and refines the upsampling model to obtain more information about the image and enhance the segmentation accuracy of small targets.

### 4.2. Fused DeepLab v3+ Model

Researchers integrated ResNet into the DeepLab *v*3+ model to improve accuracy based on the strong adaptability of the underlying network. With the improvement of the accuracy of model classification and regression, the gradual deepening of the neural network structure directly leads to the increase of the complexity of the model. In addition, the original model requires high hardware requirements, large memory consumption, and a large amount of time cost. Secondly, it is necessary to detect SiO_2_ movement state with low delay and high efficiency. Most of the production plant and equipment cannot meet the above requirements. Therefore, this paper proposes abandoning the high-complexity network architecture and integrates the lightweight neural network MobileNet v3 into the DeepLab *v*3+ segmentation model. This model retains more image features through the decoder. It also decomposes the 8-fold convolutional network into two layers and fuses the coding convolutional layer for each channel to replace the complete convolution operator. The changes to convolutional network improve the performance of the DeepLab *v*3+ decoder module to recover the boundary.

Under the premise of the same dataset, the execution time of traditional ResNet is twice that of MobileNet v3, so the network used by the model in this paper has obvious advantages in segmentation efficiency. The difference between this model and traditional model is the use of depthwise convolution; that is, each channel performs its own convolution operation with the same number of channels and filters. After the new channel feature maps are obtained, the standard 1×1 cross-channel convolution operation is performed on these new channel feature maps.

**Figure 3:**
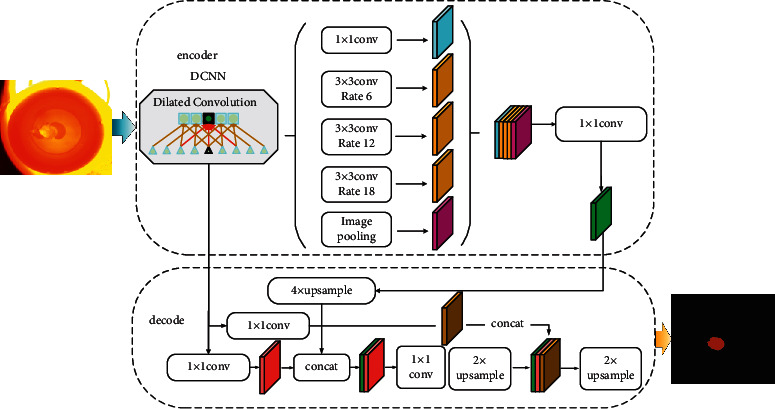
Model loss and accuracy training graph.

In Figure 3, the coding area adopts the dilated convolution structure, which extracts the features calculated by arbitrary resolution in MobileNet v3. The first is to expand the receptive field. The traditional deep network structure always uses the method of downsampling to increase the receptive field and reduce the amount of computation. Although this method can increase the receptive field, it greatly reduces the spatial resolution. Therefore, in order to prevent resolution loss, dilated convolution is adopted. The second is to capture multiscale context information; the dilated convolution can set the dilated rate (r); that is, fill *r* zeros in the convolution kernel. Therefore, when different dilated rates are set, the receptive field will be different; that is, multiscale information will be obtained. Multiscale information is very important in visual tasks. After removing the span in the last one or two blocks at the output end, an output with a stride of 16 is used to carry out more intensive feature extraction. When decoding with 8-fold stride output, compared with 16-fold stride output, the performance is improved, but the computational complexity is also increased. Therefore, a 16-fold output of 4 × 2 × 2 is used in this paper to balance the segmentation accuracy and operation speed.

### 4.3. Loss Function Improvement

The loss function used in the original model is the Cross Entropy loss function, but its biggest problem is the serious imbalance of positive and negative samples, because the negative samples (background) in the entire image account for the majority of all samples. Therefore, in the training process, the negative samples that are easy to classify will occupy the main part of the loss and affect the return of the gradient. Moreover, Cross Entropy is suitable for multiclassification sample model and is not suitable for tracking single object in this paper, which will increase the error value. So, it is necessary to improve the loss function. Inspired by X. Li [35], the method of combining loss function is adopted to alleviate the above problems. Due to different problems, different loss functions are adopted. Dice Loss comes from the Dice coefficient, a metric function used to evaluate the similarity of two samples, having a good effect on binary classification problems. The value ranges from 0 to 1. The larger the value, the more the similarity. Dice coefficient is defined as follows:

(1) $$\begin{array}{c} L_{\text{dice}}=\;\frac{2\left|X\cap Y\right|}{\left|X\right|+\left|Y\right|}, \end{array}$$

where |*X*∩*Y*| is the intersection between *X* and Y, |*X*| and |*Y*|, respectively, represent the number of elements of *X* and Y, and the numerator is multiplied by 2 to ensure that value range of the denominator after repeated calculations is between [0,1].

Therefore, Dice Loss can be written as

(2) $$\begin{array}{c} L_{\text{dice}}=1-\frac{2\left|X\cap Y\right|}{\left|X\right|+\left|Y\right|}. \end{array}$$

Dice Loss is a region-related loss. The loss and gradient value of pixel point are not only related to the label and predicted value of this point, but also related to the label and predicted value of other points, which can effectively reduce loss value. However, training loss is prone to instability, especially in the case of small targets. In addition, extreme conditions can lead to gradient saturation. Since the samples tested in this paper are too small, using this loss function will also lead to the imbalance of positive and negative samples. Therefore, Focal Loss function is introduced, which is modified on the basis of the standard Cross Entropy loss. By reducing the weight of easy-to-classify samples, the model can focus more on the difficult-to-classify samples.

(3) $$\begin{array}{c} L_{\text{Focal}}=\left\{\begin{array}{c} -\alpha {\left(1-\hat{y}\right)}^{\gamma }\text{log when}\hat{y}\;y=1\\ -\left(1-\alpha \right){\hat{y}}^{\gamma }\text{log}\left(1-\hat{y}\right)\text{when }y=0 \end{array}\right), \end{array}$$

where *α*  and *γ*  are adjustable hyperparameters and *y*=1/0 indicates that the sample is a positive sample or a negative sample. *α* ∈ [0,1], when *y*=1; the coefficient is taken as *α*, when *y* distributes different weight ratios for positive and negative samples to solve the problem of unbalanced positive and negative samples. *α* ∈ [0,1], when *y*=1; the coefficient is *α*, and when *y*=−1, the coefficient is taken as 1 −*α*. $\hat{y}$ is the target predicted value of the model, and its value is between 0 and 1. More importantly, when y=1 and $\hat{y}=1$, it represents a simple positive sample, and its contribution to the weight is 0. When *y*=0 and $\hat{y}=0$, it represents a simple negative sample, and its contribution to the weight is 0. Therefore, Focal Loss not only reduces the weight of the background class, but also reduces the weight of simple positive and negative samples. *γ*  is the adjustment of the loss function, when *γ*=0; Focal Loss is equivalent to the Cross Entropy loss function adjusted by *α*.

According to the requirements, this paper combines the advantages of these two loss functions to obtain

(4) $$\begin{array}{c} L_{D-F}=\left(1-\frac{2\left|X\cap Y\right|+\varepsilon }{\left|X\right|+\left|Y\right|+\varepsilon }\right)+\lambda L_{\text{Focal}}, \end{array}$$

where *ε* means preventing loss function from nonexistent phenomenon and *λ* is adjustment coefficient.

## 5. Analysis of Experimental Results

### 5.1. Experimental Materials, Experimental Equipment, and Experimental Procedures

The research in this paper is mainly inspired by image processing problems in high-temperature environments in the industrial production field, which is to combine the chemical industry with computer technology. It brings further improvement to the fiber-forming process of slag wool. As we all know, at high temperatures, the volume and position of high-temperature melts will change during the melting process due to Brownian motion. However, traditional image processing methods require specific brightness adjustment, regional extraction, and other preprocessing based on the acquired image information. Therefore, the efficiency in the actual production process needs to be improved. In order to reveal the dissolution behavior of iron tailings in blast furnace slag, the main component of iron tailings, silica, was used to study the melting process of silica particles at high temperatures to characterize the melting of iron ore tailing. The test used a vertical high-temperature furnace, a camera, a recording system, and a tablet press. The experimental hardware configuration was the processor AMD R7-4800H, the memory was 16 GB, the graphics card was NVIDIA GeForce RTX 3060GPU, and the operating system was Windows 10. The code compilation software uses PyCharm. The networks involved in this article were all built under the TensorFlow framework, and the experimental programming language was *Python*.

**Figure 4:**
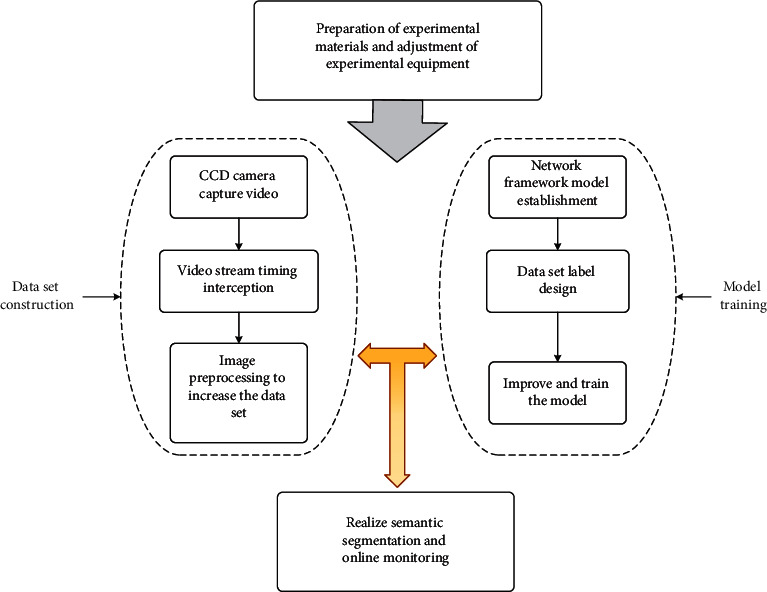
Research overall flow chart.

In order to solve the problem of lack of dataset and have good adaptability to the tracking of various bulk objects, therefore, six SiO_2_ samples with different shapes and volumes were selected, and the SiO_2_ melting process was recorded by a CCD camera, and the video stream was divided into sequence pictures with an interval of 1s, a total of 590 pictures. Using the graphical interface labeling software Labelme to label each image in the original dataset, generate multiple JSON files and finally batch converted them into grayscale images with a resolution of 224×224 and a bit depth of 24, according to the PASCALVOC data self-built database in set format. The obtained SiO_2_ pictures were expanded to 17,700 after being processed by data augmentation such as gray inversion, horizontal inversion, stretching, scaling, and rotation. In the experimental phase, the training dataset accounted for 90%, and the test dataset accounted for 10%. Transplant the MobileNet v3 network structure into the framework, replace the original Cross Entropy function with the loss function improved in this article, improve the frame of the decoding part, and finally complete all the improvements. The overall flow chart is shown in Figure 4.

In order to comprehensively select the optimal combination of dilated convolution expansion rate, compared different ASPPs are shown in Table 1. Combined different connection methods and in-depth analysis, it could be seen that the segmentation effect of the different receptive field stitching ASPP with the expansion rate combination [6, 12, 18, 24] was better than that of the combination [6, 12, 18], but the predicted consumption time for single image is 13.5% higher. The convolution group with the expansion rate combination [6, 12, 18] can increase MIoU by 0.84% and at the same time increase the prediction speed by 8.13%. Therefore, in this paper, the expansion rate of depthwise separable convolution combination of the ASPP module of different receptive fields can be selected [6, 12, 18].

**Figure 5:**
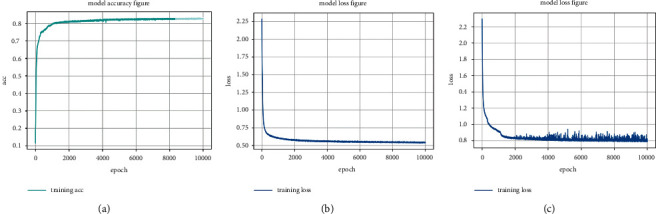
(a) The accuracy of the model in this paper fluctuates. (b) The loss of the model in this paper fluctuates. (c) The loss of the original model fluctuates.

In this paper, the optimizer chose the SGD optimization method. And to ensure that each data could be read, the batch size was set to 35, and the parameter information was updated every two samples. Extract 500 batches in one epoch, so that each sample could be extracted once, and this parameter could be updated 10,000 times. The data was saved every 200 epochs, and the segmentation accuracy changed as shown in Figure 5. Through training 10,000 times, the accuracy and loss of the model tend to be stable. The final accuracy rate was 88.8%. It could be seen from the figure that when the training is about 2000, the accuracy rate has reached more than 80%, the loss value produces a period of fluctuations and decreases rapidly, and the function converges quickly. As shown in figure (c), the loss value of the original model fluctuates violently and the final loss value is 0.7920. It shows that the loss function design in this article had an effect. For small object training, the model training was stable. This function directly calculates the error between the true value and the training value, which reduces the loss value to the greatest extent, and the final loss value is 0.6336.

### 5.2. Comparative Experiment and Performance Evaluation

In order to verify the superiority of the lightweight neural network MobileNet v3 in the segmentation model, this article compared it with the common lightweight network model. The comparison results are shown in Table 2.

In common neural network models, the higher the model depth value, the greater the number of parameters involved in the model, the more complex the model, and the greater the difficulty of training. From Table 2, the network parameters such as MobileNet v1, MobileNet v2, ShuffleNet, and Proxyless are several times that of the network MobileNet v3. In the ImageNet project, the classic ResNet50 network is more than twice the model depth of MobileNet v3. Comprehensive factors such as the highest accuracy rate, experimental hardware equipment conditions, and training time proved the necessity of choosing the lightweight neural network MobileNet v3.

However, traditional image segmentation methods, such as fuzzy C-means and watershed algorithm, simply segment images. Therefore, it is necessary to locate the pictures first, which will cause a lot of time loss and cannot be compared with the real-time tracking and segmentation of the deep learning framework.

In this paper, the MloU (mean intersection over union) and execution time were used as quantitative indicators to evaluate the segmentation accuracy and detection efficiency of the model; the engineering practicability of the model was judged based on the memory size of the generated weight file. The MIoU calculation method is as follows:

(5) $$\begin{array}{c} \text{MIoU}=\frac{\text{TP}}{\text{TP}+\text{FN}+\text{FP}},\\ \text{MIoU}=\frac{1}{k+1}\overset{k}{\underset{i=0}{\sum }}\frac{p_{\text{ii}}}{\sum_{j=0}^{k}p_{\text{ij}}+\sum_{j=0}^{k}p_{\text{ji}}-p_{\text{ii}}}. \end{array}$$

In the formula, TP represents the number of pixels that are correctly segmented into SiO_2_ regions; FN represents the number of pixels that are incorrectly marked as background SiO_2_ regions; FP represents the number of pixels that are incorrectly segmented as background. The next formula is used in the actual calculation: *p*_ij_ represents the true value of *i* and the number of predicted *j*, and *k*+1 is the number of categories (including empty categories). *p*_ij_ is the real quantity. *p*_ij_ and *p*_ij_ represent false positives and false negatives, respectively.

For the improved DeepLab *v*3+ model, the DeepLab *v*3+ basic model, FastFCN [38], and VisTR [39] tested the first 200s, the middle 200s, and the final 190s of the pictures, as shown in Table 3. It can be seen from the table that, due to the large SiO_2_ bulk in the initial melting picture, the accuracy of the three identification methods is very considerable. But starting from the mid-term, the accuracy of the object's gradual melting has decreased, and the DeepLab *v*3+ basic model has decreased significantly. In the final 190s, the melting of SiO_2_ is about to end, and the recognition accuracy of the basic model is greatly reduced. It is far inferior to the improved DeepLab *v*3+ model, which is about 6% higher. Moreover, the effect of the latest two models tested in this paper is not as good as the improved DeepLab *v*3+ model. The use of multiscale fusion of small data segmentation and binary classification loss function was well applied, and the execution time and memory consumption were only half of the original model, which fully demonstrated the advantages of lightweight network structure MobileNet v3 with low memory and high efficiency. It had little effect on accuracy. After calculation, the computational cost of the model is 0.53 B.

**Figure 6:**
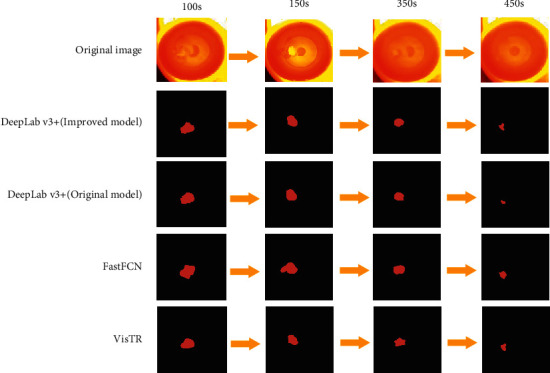
SiO_2_ comparison of different melting times.

Figure 6 is the original image at different times and the effect diagram of the original model segmentation and the model segmentation in this paper. It can be seen from the figure that the large object segmentation area of the original model is too large due to the greater influence of the interference in the furnace. For small objects of 450s, the original model segmentation area is too small, resulting in low accuracy. The reason why the proposed model had good segmentation effect for each moment was that this paper integrated convolution factors of more scales, which greatly reduced pixel value loss. Finally, the accuracy of this model was much higher than the original model, which had a good experimental application for small object tracking analysis.(Table 4)

The network structure model has certain shortcomings. The final effect of pictures with too many small objects is not ideal. The accuracy rate of the improved model in the Vaihingen dataset is only 80.6%, but the accuracy rate in the Aeroscapes dataset reaches 94.1%. Therefore, the model still needs to be adjusted and modified.

## 6. Conclusion

The original model is not accurate enough for the segmentation and extraction of small targets. Therefore, in view of the weak representation ability of detailed pixels in DeepLab *v*3+ model and the problems of missing segmentation and mis-segmentation, the relationship between each convolutional layer is further strengthened, and the multiscale fusion method was adopted to strengthen the control of the decoding layer on the details of the image. At the same time, the lightweight network was used to solve the problems of model parameter redundancy and large memory consumption, improved the running speed of image segmentation to achieve the effect of real-time monitoring, and reduced the demand for hardware. Dice Loss and Focal Loss were combined to improve the accuracy of binary classification while enhancing the weight of positive samples of small objects and reduced the fluctuation of model training and enhance the stability of the model. This model had a good effect on SiO_2_ melting motion capture, improved the control of image position and detail information, and strengthened the characterization capacity of the model. In the follow-up work, we will make an in-depth study of high-performance networks that take into account prediction accuracy and real-time performance and further enhance the practicality of semantic segmentation algorithms in engineering applications.

## Figures and Tables

**Table 1:** Comparison of test results of ASPP module improvement schemes.

Group	Dilation rate	HFS	DSAConv	MIoU/%	Training time hour	**T**_0_/**ms**
1	[6, 12, 18]			74.52	23.85	275.3
2	[6, 12, 18, 24]			74.98	25.62	310.8
3	[6, 12, 18]	√		75.39	27.37	322.4-
4	[6, 12, 18, 24]	√		75.82	30.44	372.0
5	[6, 12, 18]	√	√	75.36	21.45	253.2
6	[6, 12, 18, 24]	√	√	75.62	25.60	312.4

**Table 2:** Performance comparison of different network architectures.

Network	Top 1/%	Params (M)	MAdds (M)	CPU	Advantage
MobileNet v1	70.6	4.2	575	113 ms	Proposed depthwise separable convolution
MobileNet v2	72.0	3.4	300	75 ms	Proposed inverted residuals and linear bottlenecks
ShuffleNet(×2) [36]	73.7	5.4	524	-	Combined grouped convolution and channel shuffle
NasNet-A	74.0	5.3	564	183 ms	Designed NasNet search space
Proxyless [37]	74.6	4	320	156 ms	A new path pruning method was proposed, which reduced memory consumption
MobileNet v3	75.2	5.4	219	69 ms	Combined complementary search technology and introduced the h-swish activation function

**Table 3:** Performance comparison before and after model improvement.

Algorithm name	Pre-MIoU/%	Mid-MIoU/%	Last-MIoU/%	Time/ms	RAM/MB
DeepLabv3 + basic model	91.4	88.6	80.3	424	52
Improved DeepLabv3 + model	92.6	90.1	86.2	215	23
FastFCN	91.6	89.1	82.2	220	31
VisTR	92.4	90.1	85.2	230	41

**Table 4:** Explanation of special symbols in the text.

Symbol	Explanation	Page
**L**_**dice**_	Definition of dice coefficient	6
**X** **or** **Y**	Pixels of the whole image	6
|**X**∩**Y**|	Intersection between *X* and Y pixels	6
|**X**|**or**|**Y**|	The number of elements in *X* or Y	6
**L**_**F** **o** **c** **a** **l**_	Definition of Focal Loss function	8
*α* **or** *λ*	Tunable hyperparameters	8
$\hat{y}$	Model target predicted value	8
*ε*	Preventing nonexistence of the loss function from occurring	8
**L**_**D**−**F**_	The modified loss function definition	8
**k**	k is the number of categories (except for empty categories)	8
**M** **I** **o** **U**	Mean intersection over union	11
**p**_**ii**_	Pixels correctly segmented into SiO_2_ regions	11
**p**_**ij**_	Pixels in the SiO_2_ region that were incorrectly marked as background	11
**p**_**ji**_	Wrongly segmented into background pixels	11
**TP**	All pixels correctly segmented into SiO_2_ regions	11
**FN**	All pixels in SiO_2_ regions that were incorrectly marked as background	11
**FP**	All are wrongly segmented into background pixels	11

## Data Availability

The data used to support the findings of this study are available from the corresponding author upon request.
